# Progress in Neurology

**Published:** 1903-06-06

**Authors:** 


### Progress in Neurology.

(*Continued from paje* 155.)

**Aphasia as an Initial Symptom of Tuberculous Meningitis.**—A remarkable case has been published by Sinclair,[8] where a man, aged 28, who was suffering from chronic phthisis, suddenly developed motor aphasia and agraphia. There was no sensory impairment. The aphasia was of peculiar type, it came on in the course of ordinary conversation, as if the functional activity of the speech centre had become suddenly exhausted; this passed away after an interval of respose. This type of aphasia, which may be termed "dysphasia," resembles those cases of "dyslexia" published by Hinshelwood, where the patient becomes word-blind after correctly reading a few lines of print. The attacks of aphasia and agraphia were repeated frequently for two days, at the end of which the patient developed typical symptoms of meningitis from which he shortly died. Only two similar cases have been recorded; in those there were transient attacks of motor aphasia without agraphia, and the damage was apparently due to the tuberculous deposit affecting the commissural fibres in the island of Reil. In both cases a fatal termination ensued within a week. The tuberculous meningitis in all three cases was secondary to a tuberculous lesion existing elsewhere, either in lungs or bones.

**Rickets and Mental Enfeeblement.**—In a recent Thèse de Paris Renault[9] has made a study of the mental and cerebral conditions of rachitic children. The rachitic skull is characterised by persistence of the fontanelles and late closure of the sutures, with frontal and parietal bossing; it is large and brachycephalic. The mental condition is one of imbecility or feeble-mindedness, not one of idiocy. This mental defect is probably not congenital, but acquired as the result of gastro-intestinal or pulmonary infection. Such infection produces a rachitic alteration of the skeleton and a partial arrest in the development of the cerebral nerve-cells. The backwardness in speech and general intelligence of rachitic children is thus due to the same toxæmia which produces the rickets.

**Amaurotic Family Idiocy.**—Sachs[10] reports the results of a histological examination of the central nervous system in a typical case of this disease. There was some deficiency in the development of the cerebral white fibres and also some degeneration of the pyramidal tracts. The chief and most remarkable changes, however, were found in the larger ganglion cells throughout the entire extent of the central nervous axis. Not a normal ganglion cell could be seen, either in the cortex or the grey substance of the cord. The protoplasm of the cells had become disintegrated, leaving a more or less homogeneous mass, the nucleus had shifted to the periphery, and was not sharply differentiated from the rest of the cell. The disease thus affects the entire grey matter, and the degeneration of white matter found is a secondary phenomenon. The condition is probably due to a defective vitality of the grey matter, an example of "abiotrophy" of Gowers. In this connection it is of interest to note that similar findings have been reported in other classes of abiotrophy—in congenital spastic diplegia, in "generalised rigidity." Amaurotic family idiocy is merely a special type, which, curiously, affects chiefly children of Jewish parents.

**Tabes Dorsalis.**—In some remarks on the early recognition of tabes dorsalis, Williamson,[11] after referring to loss of knee-jerks, shooting pains, and Argyll-Robertson pupils, says that the discovery of muscular hypotonus, or loss of muscular tone, is often of value. A tabetic patient can generally perform movements to an abnormal extent at the various joints. Thus whilst a healthy person lying flat on his back cannot raise his leg, if the knee be kept fully extended, to more than an angle of 65° to 75° from the horizontal plane, a tabetic individual can raise his leg to an angle of 80°, 90°, or even 100° from the couch. This muscular hypotonus is not invariably present in tabes; further it is occasionally present in non-tabetic individuals. But in cases of suspected tabes its presence along with loss of the knee-jerk is a most valuable sign. Another little-known symptom of tabes is that there may be areas of diminished cutaneous tactile sensation on the trunk; these areas are in the form of girdles or zones round the trunk, either bilateral or unilateral. The region supplied by the mid-dorsal nerves is the part most affected. It should be tested for by the tip of the finger, warmed if necessary by hot water, so as to have the same temperature as the patient's skin. The condition of the Achillis-jerk has been investigated in a large number of persons by Bramwell,[12] who finds that it is probably constantly present in health in persons under 50 years of age; in persons over that age the activity of the jerk diminishes with increasing age, so that its absence in old people ceases to have the same diagnostic significance that it has at an earlier age. In the great majority of cases of tabes both the knee-jerks and the Achillis-jerks are absent, but in some cases of early tabes the absence of the Achillis-jerks has been noted whilst the knee-jerks were still present. This may be of considerable importance in diagnosis. The best method of eliciting the jerk is for the patient to kneel on a chair with his feet projecting a few inches over the edge. The tendo-Achillis is then struck sharply with a hammer of some kind close to its insertion, when the jerk is shown by a quick plantar flexion of the foot at the ankle joint.

**Postero-lateral Scleroses.**—Burr and McCarthy,[13] after describing the pathology of several cases of combined system disease of the cord, say that for the present we may most conveniently classify such cases as follows :—(1) Friedreich's ataxy ; (2) tabes with associated diffuse sclerosis extending into the lateral columns ; (3) tabes with degeneration in the crossed pyramidal tracts and also in the direct cerebellar tracts ; (4) posterior sclerosis with sclerosis of the lateral columns and disease of the anterior horns ; (5) primary lateral sclerosis with minor changes in the posterior columns ; (6) subacute diffuse degeneration of the spinal cord due to anæmia, cachexia, sepsis, etc., with sclerosis in the posterior and lateral tracts ; (7) diffuse interstitial sclerosis seen occasionally in chronic alcoholism with multiple neuritis, and in which the degeneration is secondary to overgrowth of glial and connective tissue elements ; (8) a system disease of unknown origin distinctly confined to the direct and crossed pyramidal tracts and the posterior columns.

**Transverse Lesions of the Spinal Cord.**—A critical review of the bearing of recently-reported cases on Bastian's view that a complete lesion of the cord is accompanied by flaccidity of the limbs and loss of the tendon reflexes, is published by Warrington.[14] These cases show that whilst Bastian's law is generally true, yet when the disease is of a slowly-progressive nature the reflex functions of the cord may be retained and the muscular tone be unimpaired, and also that in some cases very severe symptoms approximating to those generally found after complete transverse lesions may be present and yet the anatomical lesion be slight or incomplete. This is especially observed in compression of the cord and indicates that physiological loss of function may be produced without marked anatomical change. From the point of view of surgical interference it is important to notice that no case of incomplete division of the cord has been recorded in which all the symptoms described by Bastian were present and in which the lumbar enlargement was intact. The explanation of these facts is not yet certain. Bastian based his explanation on the hypothesis of Hughlings Jackson on the antagonism between cerebral and cerebellar influence. When the cerebrospinal system is destroyed, this permits unrestrained action of the cerebellar influence and there results increase of muscle tone and of the tendon phenomena. When both are cut off there results flaccidity of the muscles with loss of tone. Recent researches on the anatomical connections of the brain and spinal cord have but slightly modified this view, so that at present the theory may be stated as follows :—(1) The motor-cortex exercises an inhibitory influence by means of the cortico-spinal tract. (2) The cortex of the cerebellum and grey masses of the mesencephalon and rhombencephalon transmit an exciting influence to the neurones of the spinal cord by means of the cerebello-spinal fibres and mesencephalic tracts. If a lesion of (1) occurs there results an increase of tonus and of reflexes. If both (1) and (2) are cut a diminished tonus and reflexes follow. The long efferent mesencephalic fibres are thus especially concerned in the maintenance of muscular tone and of the tendon reflexes.

**Acute Myelitis.**—Singer[15] maintains that so-called acute myelitis is found on microscopical examination in the majority of recorded cases to be not inflammatory but due to thrombosis of spinal vessels. The most common cause of this is syphilitic arteritis ; senile arterial degeneration accounts for a considerable proportion of the remaining cases. This view is confirmed clinically by the analogy between this disease and cerebral thrombosis. This view is of practical value, for it is possible by prompt treatment, in many cases presenting premonitory symptoms and giving a history of recent syphilis, to ward off an attack of this nature. For this reason alone it would be better, instead of using the term "myelitis" to classify such cases as "thrombotic softening of the spinal cord," or more simply as "spinal thrombosis."

**Lesions of the Brachial Plexus.**—Six cases of lesion of one of the spinal nerves forming the brachial plexus have been recorded by Buzzard.[16] The objective disturbances of sensibility resulting from this lesion closely correspond to those obtained by Sherrington after cutting a posterior root in a monkey, and differ in some respects from the anæsthesia produced by division of a peripheral nerve. The anæsthesia is largely dissociative in character and does not occupy the whole cutaneous area to which the afferent fibres of the spinal nerve are distributed ; it is not associated with any subjunctive sensation of numbness, and is consequently overlooked by the patient. The atrophy of the affected muscles is in excess of what might be expected in view of the fact that many of the muscles receive fibres from two or more spinal nerves. The morbid process is probably vascular in character and may be sudden or gradual in its onset.

[8] Brit. Med. Jour., Dec. 20, 1902. [9] Lancet, Jan. 10, 1903. [10] Jour. of Nerv. and Ment. Dis., Jan. 1903. [11] Med. Chron., Aug. 1902. [12] Scot. Med. and Surg. Jour., Dec. 1902. [13] Jour. of Nerv. and Ment. Dis., Jan. 1903. [14] Med. Chron., Nov. 1902. [15] Brain, 1902. [16] Ibid.

*(To be continued.)*

